# Supplementary material for: Effect of Probiotic Lactobacillus plantarum on Streptococcus mutans and Candida albicans Clinical Isolates from Children with Early Childhood Caries
Source: Int J Mol Sci. 2023 Feb 3;24(3):2991. doi: 10.3390/ijms24032991 (PMC9917570; doi:10.3390/ijms24032991)
Supplement: Supplementary file 1 [file ijms-24-02991-s001.zip › ijms-2168816-supplementary.pdf]

Title:

Effect of probiotic *Lactobacillus plantarum* on *Streptococcus mutans* and *Candida albicans* clinical isolates from children with early childhood caries

Supplementals

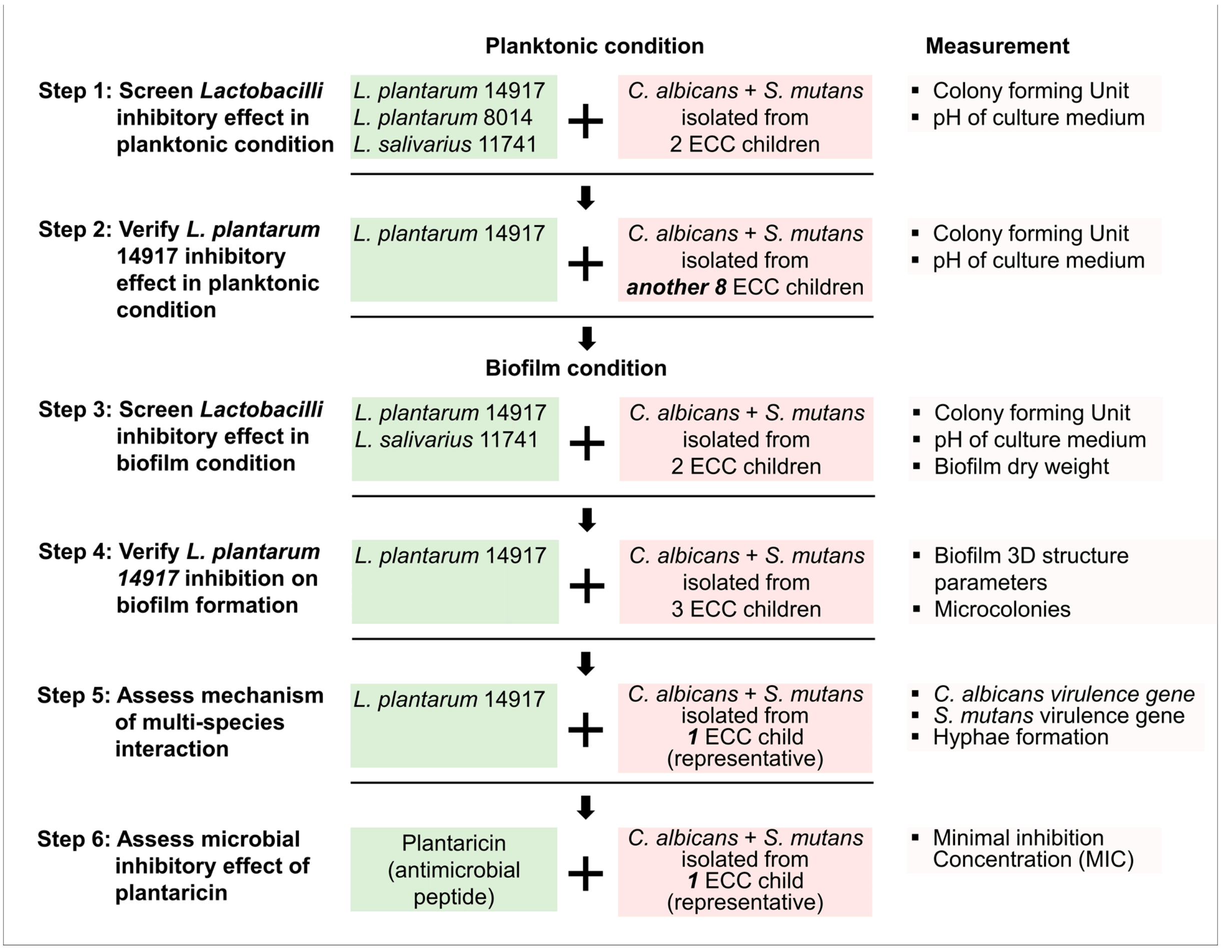

Figure S1. Study design

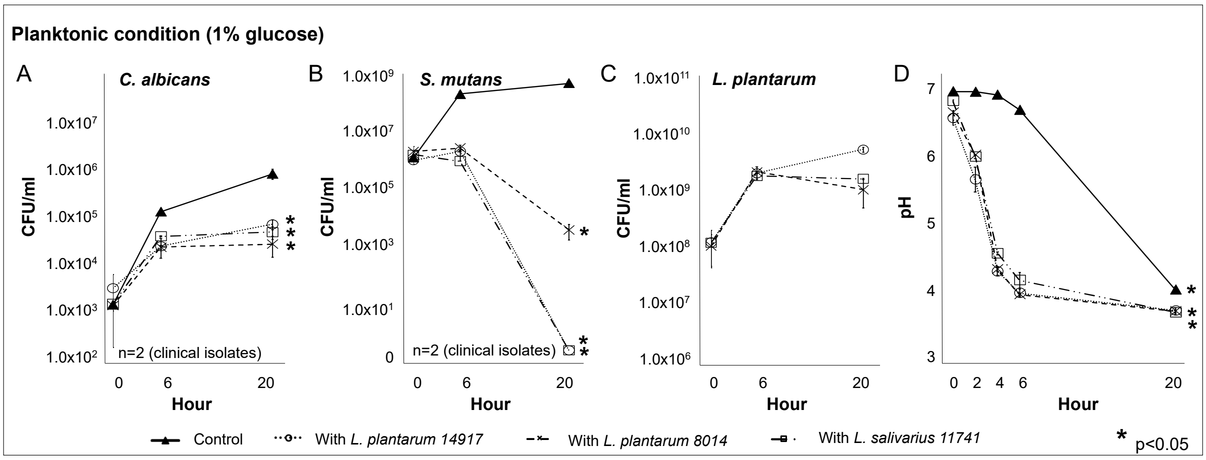

Figure S2. Inhibitory effect of *Lactobacillus species* on clinically isolated *C. albicans* and *S. mutans* from children with early childhood caries in multispecies planktonic condition.

The growth of *C. albicans*, *S. mutans,* and *Lactobacillus spp.* in multispecies planktonic condition are plotted. *C. albicans* and *S. mutans* clinical strains were isolated from two children with early childhood caries (ECC). Experiments repeated in triplicates. Each planktonic multispecies condition included the *C. albicans* and *S. mutans* isolated from the same ECC child, with added *L. plantarum* 14917. The control group only consisted of *C. albicans* and *S. mutans*. The treated group included *Lactobacilli* was marked as "with *Lactobacillus spp.*". (A) All three *Lactobacillus* inhibited the growth of *C. albicans* by <1 log after 6 hours and 1-2 logs following 20 hours' incubation. (B) All three *Lactobacillus* inhibited the growth of *S. mutans*. The performance is ranked by *L. plantarum* 14917 and *L. salivarius* 11741, and *L. plantarum* 8014. All three *Lactobacilli* significantly inhibited the growth of *S. mutans* at 6 hours by 2 logs. *L. plantarum* 14917 and *L. salivarius* 11741completely inhibited the growth of *S. mutans* after 20 hours, with the exception of *L. plantarum 8014*. (C) *Lactobacilli* maintained a stable growth during the 20 hours' interaction with *S. mutans* and *C. albicans*. (D) The culture medium pH dropped rapidly with the addition of *Lactobacilli* in planktonic condition, both control and treated groups reached the same acidic level at 20 hours.

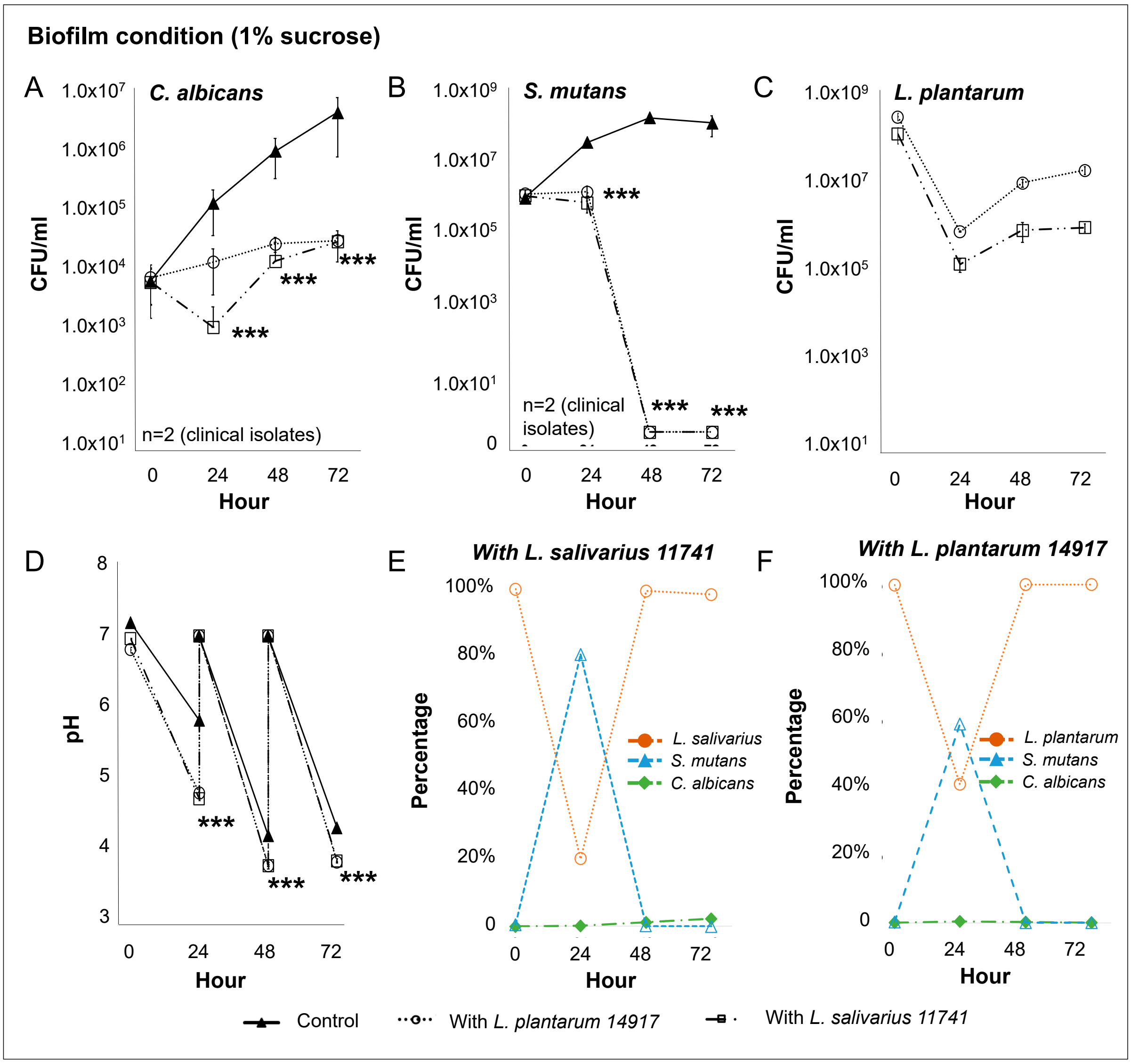

Figure S3. Inhibition of *C. albicans* and *S. mutans* in ECC children by *Lactobacilli* in multispecies biofilms.

Multispecies biofilms were formed by *L. plantarum* 14917 and *L. salivarius* 11741 and clinically isolated *C. albicans*, *S. mutans* from two children with ECC. The control group consists of *C. albicans* and *S. mutans* from the same ECC child. (A) *L. plantarum* and *L. salivarius* inhibited the growth of *C. albicans* by 3 logs compared to the control group. (B) At 48 hours, after two times administration of *L. plantarum* and *L. salivarius*, *S. mutans* was completely inhibited in biofilms of treatment group. (C) *Lactobacilli* maintained stable growth during 72-h incubation period with *L. plantarum* being significantly higher than *L. salivarius.* (D) The pH of the culture medium was significantly lower with added *Lactobacilli* at 24, 48 and 72 hours, comparing to the control group ($p<0.05$). (E-F) The composition of each microorganism is plotted. *Lactobacilli* became the dominate species after 48 h incubation.

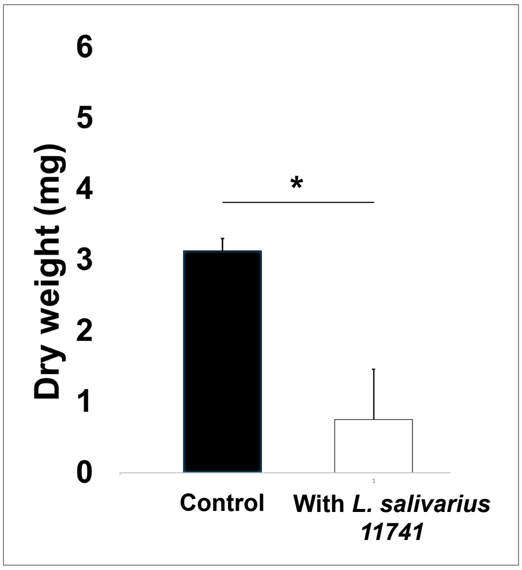

Figure S4. Dry weight of biofilms treated by *L. salivarius*

Multispecies biofilms were formed by *L. salivarius* 11741 and clinically isolated *C. albicans*, *S. mutans* from two children with ECC. The control group consists of *C. albicans* and *S. mutans* from the same ECC child. Biofilm formation was reduced with *Lactobacilli*, almost 70% reduction with *L. salivarius* in term of measurement by the dry-weight at 72 hours.

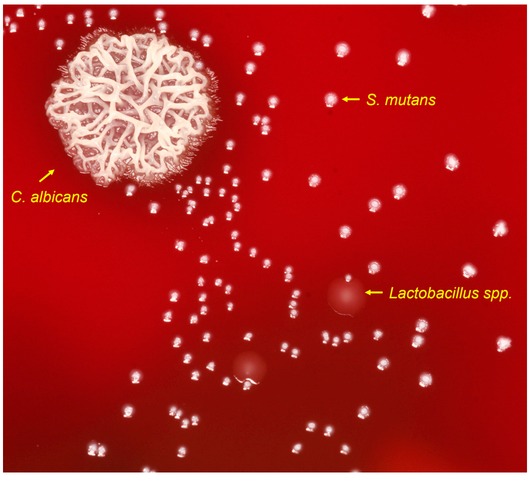

Figure S5. Distinct morphological differences between *S. mutans*, *C. albicans*, and *Lactobacillus* spp.
